# Oncolytic virotherapy with SOCS3 enhances viral replicative potency and oncolysis for gastric cancer

**DOI:** 10.18632/oncotarget.27873

**Published:** 2021-02-16

**Authors:** Shuichi Matsumura, Mikihito Nakamori, Toshiaki Tsuji, Tomoya Kato, Masaki Nakamura, Toshiyasu Ojima, Hiroshi Fukuhara, Yasushi Ino, Tomoki Todo, Hiroki Yamaue

**Affiliations:** ^1^Second Department of Surgery, Wakayama Medical University, Wakayama, Japan; ^2^Department of Urology, School of Medicine, Kyorin University, Tokyo, Japan; ^3^Division of Innovative Cancer Therapy, The Advanced Clinical Research Center, The Institute of Medical Science, The University of Tokyo, Tokyo, Japan; ^4^Division of Digestive Surgery, Osaka Minami Medical Center, National Hospital Organization, Kawachinagano, Osaka, Japan

**Keywords:** oncolytic virus, herpes simplex virus, gastric cancer

## Abstract

Oncolytic virotherapy is an encouraging treatment using herpes simplex virus (HSV) for gastric cancer patients. To treat gastric cancer, we generated and evaluated the efficacy of an attractive type of oncolytic HSV expressing the suppressor of cytokine signaling 3 (SOCS3). We constructed a third-generation type of oncolytic HSV (T-SOCS3) arming with SOCS3 by a bacterial artificial chromosome (BAC) system. We examined the viral replicative intensification and oncolysis of T-SOCS3 for human gastric cancer cell lines *ex vivo*. T-SOCS3 enhanced its replication and potentiated its cell-killing effect for MKN1 human gastric cancer cell lines, which are resistant to a non-armed third-generation type of oncolytic HSV (T-01) *ex vivo*. T-SOCS3 also induced the destruction within human gastric cancer specimens. Armed oncolytic HSVs expressing SOCS3 may be an efficacious therapeutic agent for gastric cancer treatment.

## INTRODUCTION

Gastric cancer is currently the world’s fifth ordinary cancer and the third most common cause of death. Standard treatment available for advanced gastric cancer is surgical resection combined with appropriate lymph node dissection. When the cancer is unresectable, however, cytotoxic chemotherapy is generally used. Molecular target drugs and immune checkpoint inhibitors such as ramucirumab [1] and nivolumab [2–4] are also used, but there are few treatment options for unresectable or recurrent gastric cancer with progression after standard treatment. The prognosis of patients with liver or distant lymph node metastases is also relatively poor, similarly to scirrhous gastric cancer with cancer dissemination in the peritoneal cavity. Therefore, novel therapeutic innovations for advanced or recurrent gastric cancers are strongly desired.

Genetically engineered onclytic viruses (OVs) can infect cancer cells. After infection, OVs can only replicate in cancer cells and induce viral oncolysis [5–10]. Among many types of OVs, oncolytic herpes simplex viruses (oHSVs) have raised the possibility for clinical application of oncolytic virotherapy [11]. In practice, phase I, II and III clinical investigations using some oHSVs (such as G207, NV1020 and OncoVex^GM-CSF^) for patients with a variety of solid cancers have already conducted [12–15]. Oncolytic virotherapy for gastric cancer, meanwhile, is not widely reported [16, 17]. We have previously reported that oHSVs expressing thrombospondin-1 (TSP-1) enhanced the efficacy of oncolytic HSVs for human gastric cancer cell lines, and oHSVs expressing TSP-1 suppressed gastric cancer cell proliferation both *ex vivo* and *in vivo* [18]. However, we also found that oHSVs expressing TSP-1 did not induce cytotoxicity to all types of gastric cancer cell lines, and some cell lines, such as MKN1, were resistant to viral treatment. We suggest the need to develop novel oHSVs treatment based on the biological properties between viruses and gastric cancer cells.

In this study, we turned our attention to the suppressor of cytokine signaling (SOCS) family which is an important regulator of cytokine signals [19]. Among SOCS family proteins, SOCS3 plays an important role of cytokine signal control in Janus kinase (JAK)-signal transducer and activator of transcription (STAT) signaling. JAK-STAT signaling pathways are mainly associated with extracellular stimulation by cytokine family including interleukin (IL)-6, granulocyte colony stimulating factor (G-CSF), and interferon (IFN)-γ. JAK-STAT signaling pathways also regulate pleiotropic property of cellular differentiation, maturation, and migration [19]. Furthermore, SOCS3 has often been identified to be silenced in various cancer cells owing to hypermethylated DNA at the domain of CpG island in its functional promoter region [20]. Based on the above reasons, we hypothesized that SOCS3 restoration in gastric cancer cells could suppress STAT activity and regulate cancer cell amplification through a SOCS3 gene delivery approach.

More importantly, SOCS3 is also induced from an early stage of HSV type 1 (HSV-1) infection and it suppresses the phosphorylation of the STAT system by IFN-γ [21]. In this regard, SOCS3 creates a favorable environment for intracellular replication of HSV-1. Association between HSV-1 and SOCS3 has been reported. HSV-1 inhibits IFN by expressing SOCS3. Inhibiting SOCS3 induction during HSV-1 infection releases the IFN signaling pathway and suppresses HSV-1 replication [22]. We hypothesized that oHSVs expressing SOCS3 can effectively replicate and destroy within gastric cancer cells.

Here, we focus on SOCS3 for oncolytic virotherapy using HSV for gastric cancer. We generated a new type of oHSVs expressing SOCS-3 (T-SOCS3) and compared with the third-generation type of oncolytic HSV (T-01) with T-SOCS3 in viral replication potency and the oncolytic effect.

## RESULTS

### *Ex vivo* SOCS expression and cell-killing effect after T-01 infection for human gastric cancer cell lines

**Figure 1:**
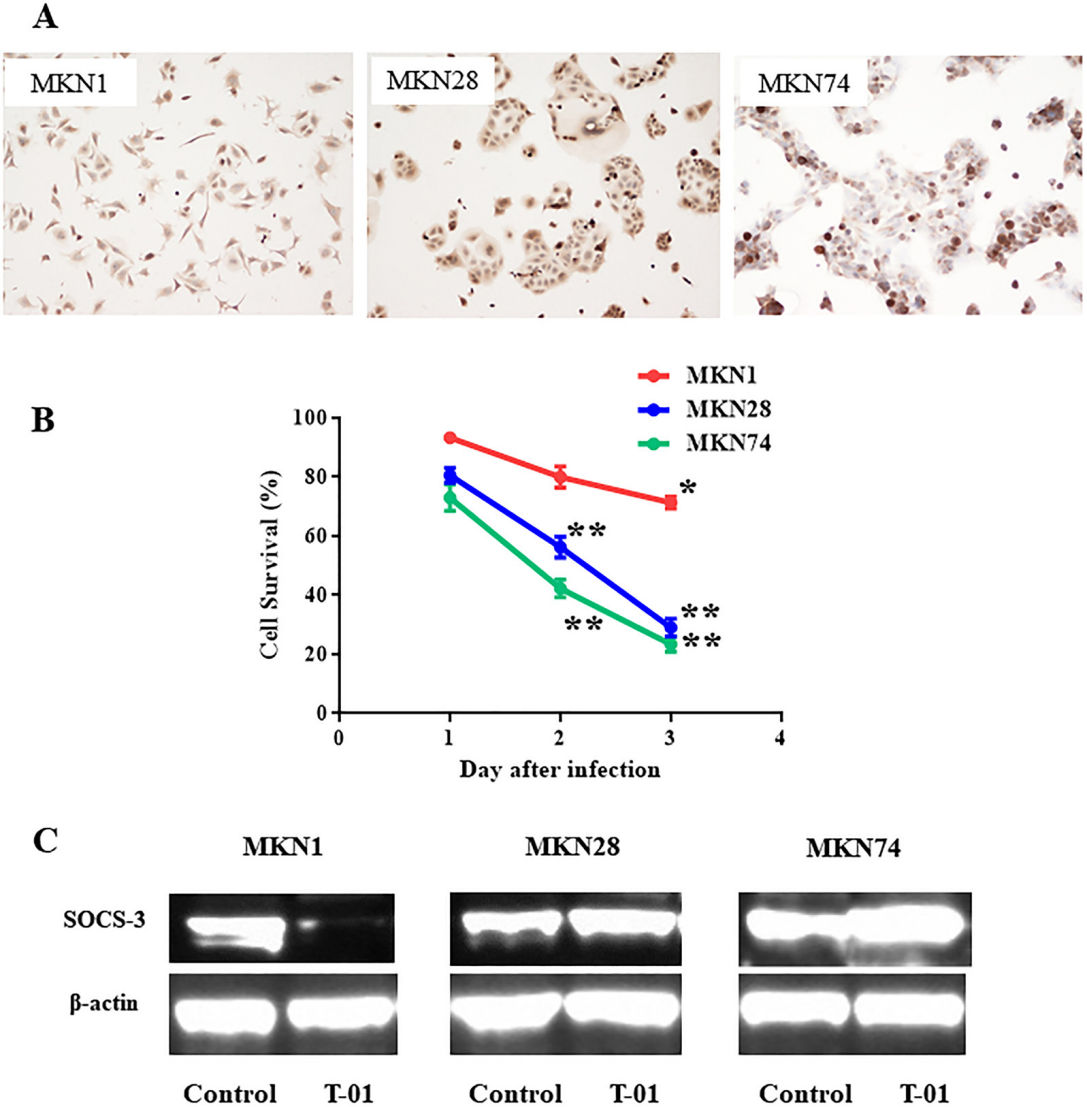
SOCS3 expression in human gastric cancer cell line after oncolytic virus infection. (**A**) Immunocytochemical staining for MKN1, MKN28, and MKN74. (**B**) T-01 was used to treat gastric cancer cell lines *ex vitro*. MKN1, MKN28, and MKN74 cells were seeded on 24 well plates at 1 × 10^4^ per well and incubated. After 24 hr of incubation, the cells were infected through T-01 at MOI of 0.01 and further incubated at 37°C. The number of viable cancer cells were measured by the amount of LDH from cancer cells using a the CytoTox 96 assay (Promega, WI, USA) according to the instruction manual, and the cell viability was expressed as a percentage of the PBS (–) treated control cells. LDH measurement was performed in triplicate wells. Data are presented as the mean of triplicates ± SD. One-way ANOVA followed by a Dunnett’s test was used to determine statistical significance (^*^ *p* < 0.05, ^**^ *p* < 0.01; versus PBS (–) treated control cells). (**C**) MKN1, MKN28, and MKN74 human gastric cancer cells were seeded in 10-cm dish at 2 × 10^6^ cells per dish and incubated at 37°C. After 24 hr of incubation, cells were infected through PBS (–) and T-01 (at MOI of 0.1) and incubated further at 39.5°C for 20 hr and harvested. Proteins (30 μg) were applied to sodium dodecyl sulfate polyacrylamide gel electrophoresis (SDS-PAGE), transferred to nitrocellulose membrane, and blotted for 2 hr with monoclonal mouse anti-SOCS3 antibody or for an hour with mouse anti-b-actin antibody (diluted 1:2000, Sigma).

To confirm the molecular expression of SOCS3 in human gastric cancer lines before viral infection, we performed immunocytochemical staining with an anti-human SOCS3 antibody. SOCS3 expression was detected in human gastric cancer cell line (MKN1, MKN28, and MNK73) (Figure 1A). As previously reported [18], T-01 is a second-generation type of oHSV, generated from deletion of the ICP6 gene, α47 gene, and both copies of the γ34.5 gene. T-01 could induce cell-killing effect against MKN28 and MKN73 gastric cancer cell lines, but MKN1 cells were treatment-resistant to T-01. At 48 hr after T-01 infection at multiplicity of infection (MOI) of 0.01, 76% of MKN28 and 81% of MKN74 induced cell-killing via viral oncolysis. In contrast, only 17% of the MKN1 cells induced cell-killing by T-01 (Figure 1B). To determine the expression of SOCS3 after infection of T-01, MKN1, MKN28, and MNK74 gastric cancer cell lines were transmitted to T-01 at MOI of 0.01. As a results of Western blot analysis, the expression of SOCS3 was detected in MKN28 and MKN74 cells after T-01 infections did not decrease the expression of SOCS3. On the other hand, the expression of SOCS3 in MKN1 gastric cancer cells was suppressed by T-01 infection (Figure 1C). These results suggest that T-01 could reduce the viral replication via downregulation of SOCS3 in MKN1 gastric cancer cells. In this context, we further examined the cell-killing effect and a viral replication assay of T-01 or T-SOCS3, in a minimally sensitive human gastric cancer cell line, MKN1.

### Generation of an oncolytic herpes simplex virus expressing SOCS3

**Figure 2:**
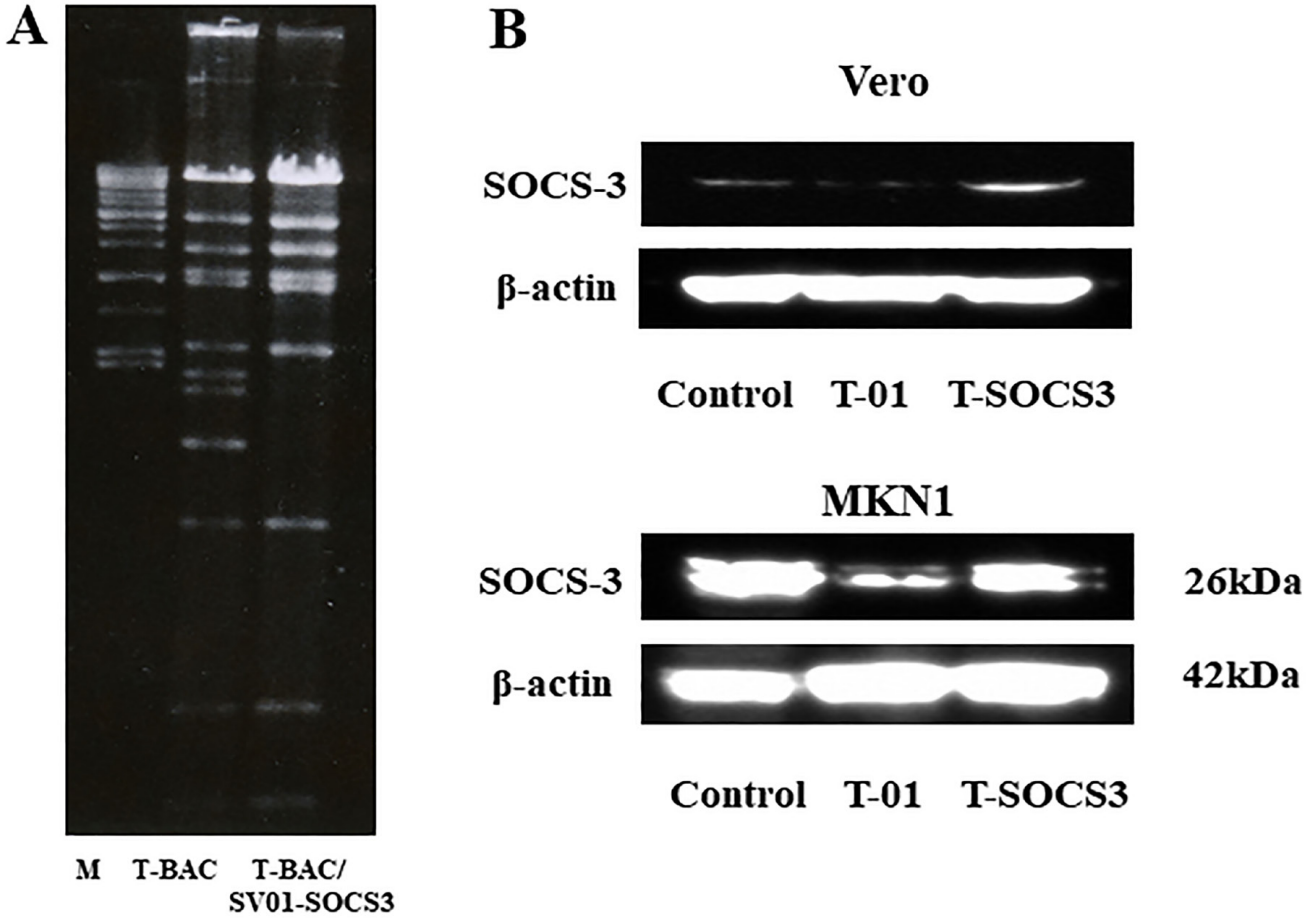
Verification of T-SOCS3 expression in oncolytic HSV-1-infected Vero cells. (**A**) BAC plasmids were digested with *Hind* III. The digested BAC plasmids were electrophoresed, T-BAC (left) and Cre-recombinant BAC plasmid, T-BAC/SV-SOCS3 (right). (**B**) Western blotting for Vero cells and MKN1 cells infected with T-SOCS3 (at MOI of 0.01) with an anti-SOCS3 antibody.

To generate an oHSV expressing human SOCS3 gene, which we designated as T-SOCS3, we used combined methods with a bacterial artificial chromosome (BAC) and Cre-loxP and FLPe-FRT recombinase method as previously reported [18, 23]. At first, the SOCS3 gene was ligated into the multicloning site of the shuttle vector SV-01, and then the SOCS3 expressing vector was generated and designated as SV-SOCS3. Second, to confirm the insertion of SV-SOCS3, the recombinant BAC plasmid (T-BAC/SV-SOCS3) or T-BAC alone were then performed restriction enzyme analysis with *Hind III* and electrophoresed as previously reported [18, 23]. Insertion the cassette of both the SOCS3 gene driven from a cytomegalovirus (CMV) promoter and the lacZ gene as a marker into the deleted ICP6 locus was reconfirmed (Figure 2A). The viral property of T-SOCS3 is the same as T-01 which had deletions of the ICP6 gene, α47 gene, and both copies of the γ34.5 gene.

### *Ex vivo* western blot analysis after infection of T-SOCS3

**Figure 3:**
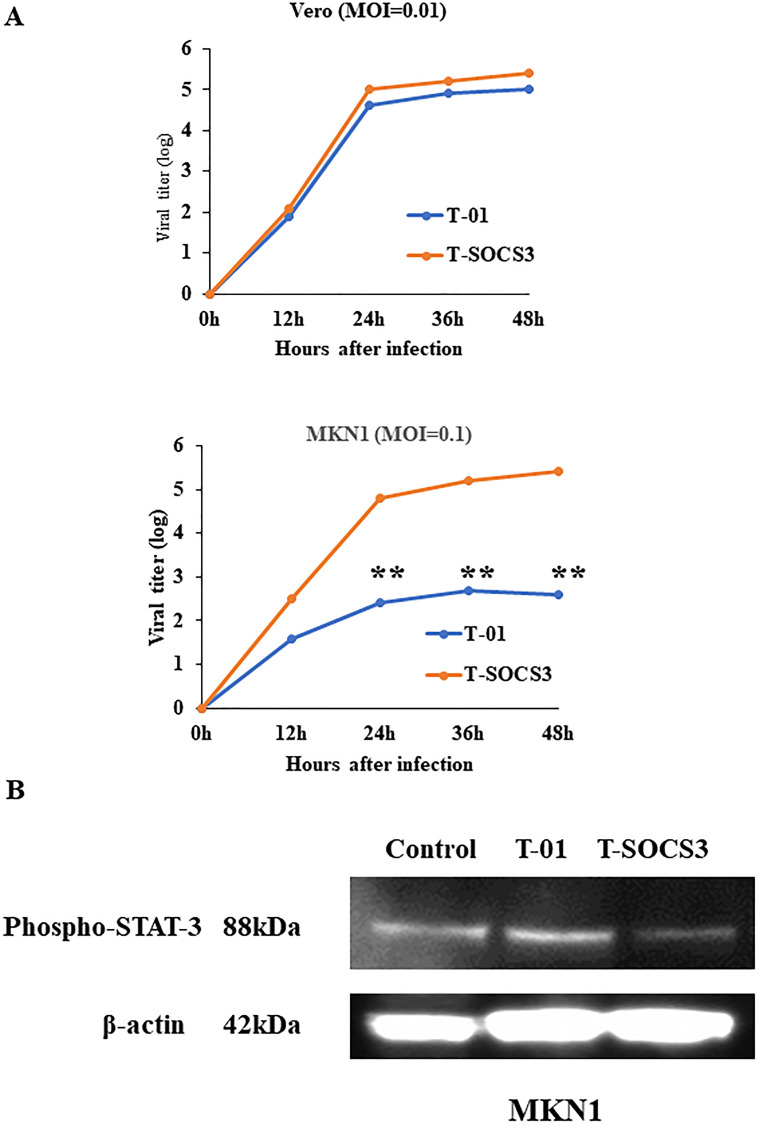
*Ex vivo* viral replication property of T-SOCS3 for Vero cells and MKN1 gastric cancer cell lines. (**A**) Vero cells and MKN1 cells were seeded into 6 well plates at a density of 5 × 10^5^ per well. Each well was infected through either T-01 or T-SOCS3 at MOI of 0.01 (for MKN1 cells, at MOI of 0.1) and the viral replication was determined. The viral titer of T-SOCS3 had an approximately 20-fold enhanced ability to replicate relative to T-01 in MKN1 cells (^**^ *p* < 0.01). (**B**) MKN1 human gastric cancer cells were seeded in 10-cm dish at 2 × 10^6^ cells per dish and incubated at 37°C. After 24 hr of incubation, cells were infected through PBS (–) and T-01 (MOI of 0.1) and incubated further at 39.5°C for 20 hr and harvested. Proteins (30 μg) were applied to sodium dodecyl sulfate polyacrylamide gel electrophoresis (SDS-PAGE), transferred to nitrocellulose membrane, and blotted for 2 hr with monoclonal mouse anti-phosphorylated-STAT3 (pSTAT3) antibody or an hour with mouse anti-b-actin antibody (diluted 1:2000, Sigma).

To confirm the activity of an oHSV encoding SOCS3 gene (T-SOCS3), Vero cells were infected through T-01 (at MOI of 0.01), T-SOCS3 (at MOI of 0.1), or PBS (–) as control. MKN1 cells were also infected through T-01 (at MOI of 0.01), T-SOCS3 (at MOI of 0.1), or PBS (–) As a result of Western blot analysis, infection of T-SOCS3 led to increased expression of SOCS3 increased in MKN1 cells (Figure 2B). By contrast, the expression of SOCS3 in MKN1 cells was suppressed by T-01. In addition, the expression of phosphorylated STAT3 (pSTAT3) in MKN1 cells was found to be suppressed by T-SOCS3 (Figure 3B). These results suggest that T-SOCS3 could control the activity of JAK-STAT3 signaling pathway in MKN1 cells, which is resistant to conventional oHSVs such as T-01.

### *Ex vivo* comparison of viral oncolysis and replication of T-01 and T-SOCS3 in MKN1 human gastric cancer cell lines

**Figure 4:**
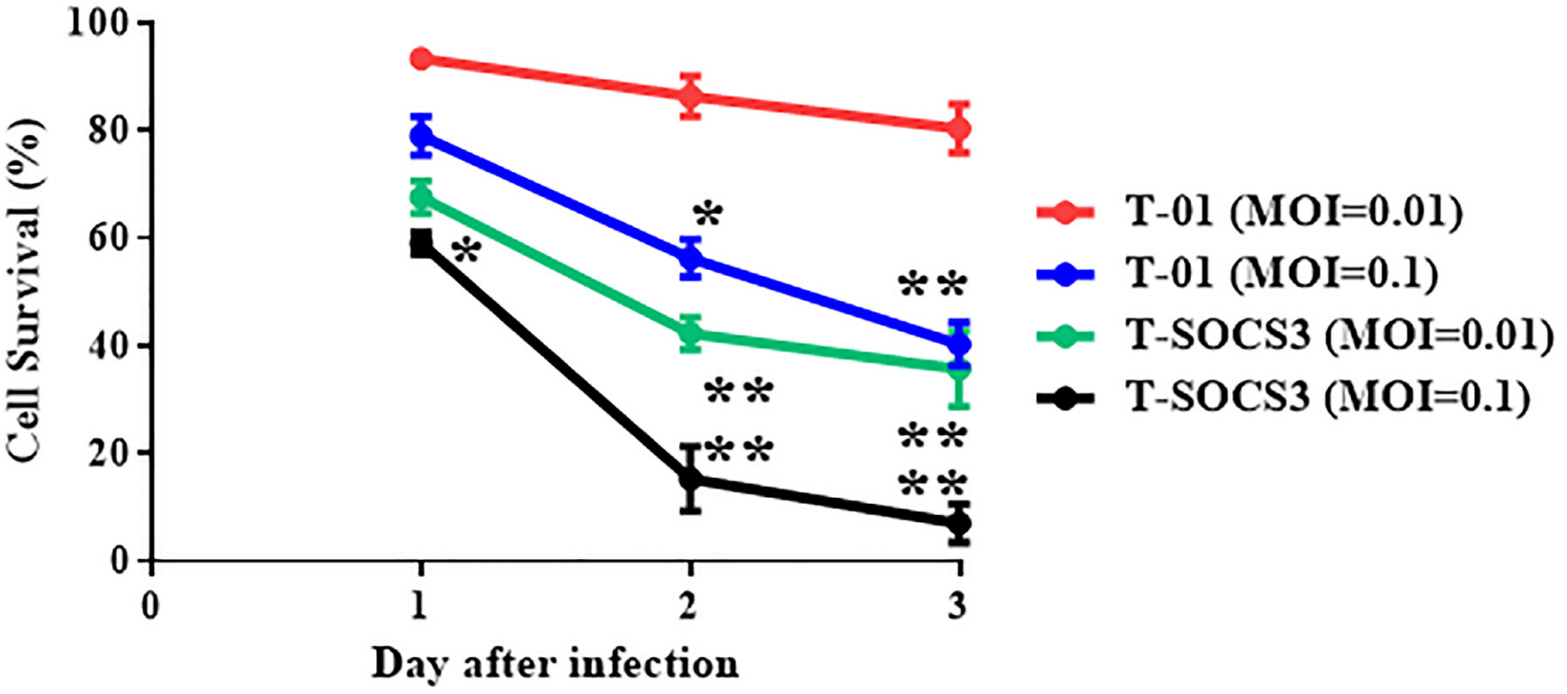
Comparison of cell-killing effect of T-01 and T-SOCS3 *ex vivo*. For cell-killing studies of T-01 and T-SOCS3, cells were seeded on 24-well plates at 1 × 10^4^ per well and incubated for 24 hr. Each well was infected through either T-01 or T-TSP-1 at MOI of 0.1 or 0.01, and further incubated at 37°C. The number of viable cells was measured daily and was expressed as a percentage of the PBS (–) treated control. Data are presented as the mean of triplicates ± SD. One-way ANOVA followed by a Dunnett’s test was used to determine statistical significance (^*^ *p* < 0.05, ^**^ *p* < 0.01; versus PBS (–) treated control cells).

At 72 h after viral infection, T-SOCS3 also showed excellent cell-killing effects compared with T-01 at MOI of 0.1 (Figure 4). We compared the replication potencies of T-SOCS3 with those of T-01 in Vero cells and MKN1 cells. The results showed that the viral titer of T-SOCS3 had an approximately 20-fold enhanced ability to replicate relative to T-01 in MKN1 gastric cancer cells (Figure 3A).

### *Ex vivo* effect of T-SOCS3 on gastric cancer specimen tissue in collagen gel culture

**Figure 5:**
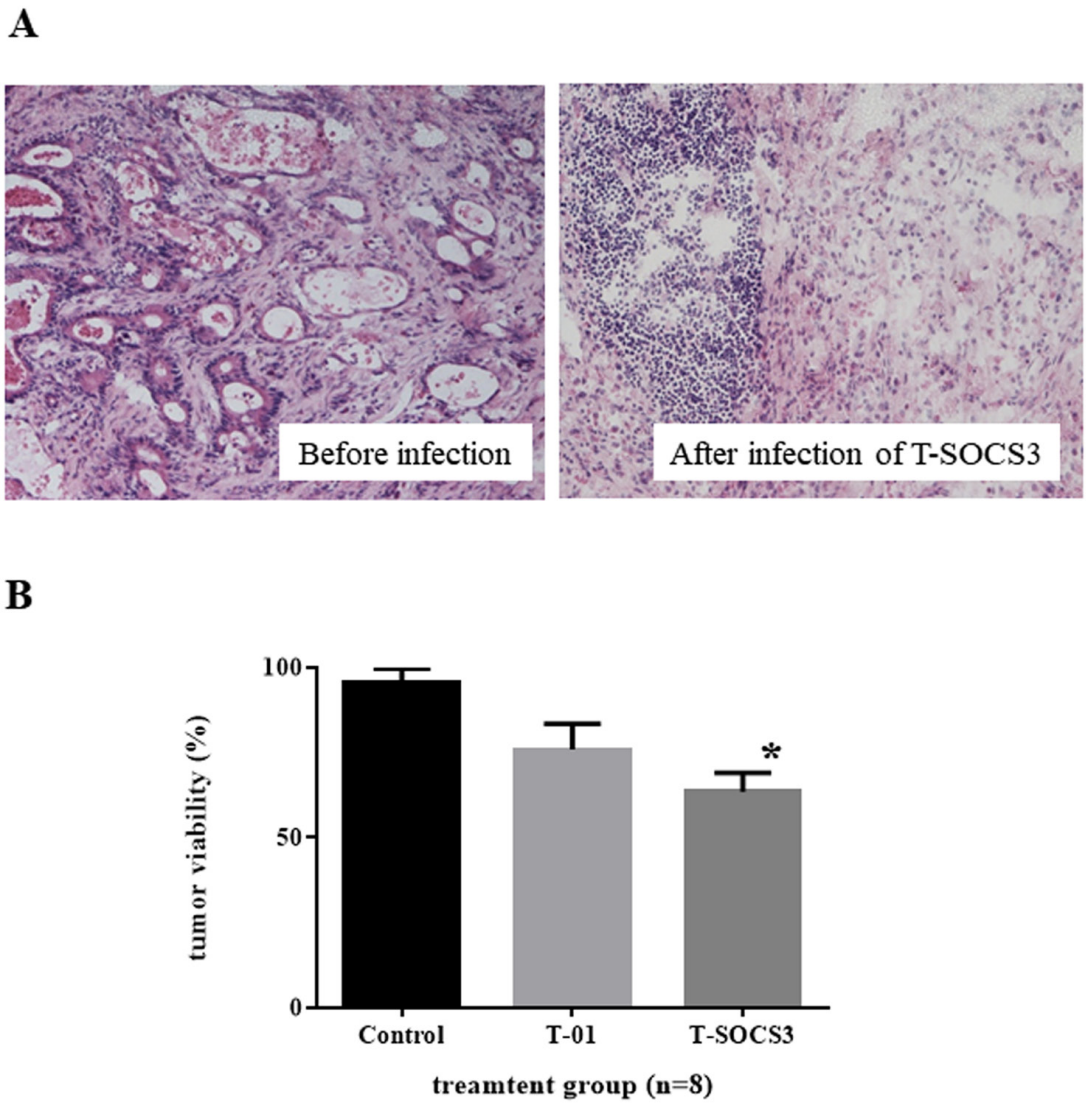
*Ex vivo* assessment of oncolytic herpes simplex virus killing effect in gastric cancer. (**A**) Gastric cancer specimens treated with T-SOCS3 (*left*, before infection, *right*; 72 hr after infection). (**B**) At 72 hr after infection through T-01 or T-SOCS3 at 0.01 pfu/cell, 25.2% and 36.5% of cells had been killed, respectively. The T-SOCS3 treatment group showed significantly lower cell viability in comparison with both the control group and T-01 group. Data are presented as the mean of triplicates ± SD. One-way ANOVA followed by a Dunnett’s test was used to determine statistical significance (^*^ *p* < 0.05: versus PBS (–) treated control tumor tissues).

To examine the effects of oHSVs in gastric cancer *ex vivo*, in terms of experimental animal protection and management, human gastric adenocarcinoma specimens derived from radical gastrectomy were incubated *ex vivo* on collagen gel immediately after surgical resection and were infected with PBS (–), T-01, or T-SOCS3. At 72 hr after infection, these specimens underwent frozen sectioning and were observed through hematoxylin and eosin (H&E) staining. In the gastric cancer specimens with oHSV infection, oncolysis was observed in the tumor tissues (Figure 5A). According to pathological confirmation by gastroenterological pathologist, there was no difference in the cellular or tissue destruction between the T-01 treatment group and T-SOCS3 treatment group. At 72 hr after infection with T-01 or T-SOCS3 at 0.01 pfu/cell, 25.2 and 36.5% of cells had been killed, respectively. The T-SOCS3 treatment group demonstrated significantly lower cell viability in comparison with the control PBS (–) treatment group. As such, compared with T-01, T-SOCS3 did show significant cytotoxicity (Figure 5B).

## DISCUSSION

Virotherapy has been suggested to have great potential as a treatment platform for improvement of prognosis in patients with advanced cancer. One of its advantages is the lack of cross resistance with the standard therapies used for adjuvant treatments such as chemotherapy, molecular target therapy, and immune checkpoint inhibitors. Talimogene laherparepvec (T-VEC), a second-generation type of oHSV expressing granulocyte macrophage colony-stimulating factor (GM-CSF), have received the first approval of the US Food and Drug Administration (FDA) for advanced melanoma patients [24].

G47Δ is a triple gene-mutated, third-generation type of oHSV [25]. This oHSV was generated from another deletion mutation to the genome of a second-generation type of oHSV, G207. G47Δ exhibited potent antitumor efficacy in preclinical studies, while retaining acceptable safety profile [26, 27]. For refractory glioblastoma patients, the first-in-human clinical phase I trial (UMIN000002661) in Japan for r demonstrated safely intratumoral administration of G47Δ. Moreover, enrollment of the subsequent phase II clinical trial (UMIN000015995) has also just finished and come out expected results. Our collaborators have also recently reported the preclinical therapeutic evaluation by G47Δ intratumoral administration in an orthotopic gastric cancer model, which mimics the clinical presentation of refractory scirrhous gastric cancer [28]. In the current study, we generated a newly designed oHSV expressing SOCS3 that has the same genetic backbone of G47Δ.

We previously reported that oHSVs expressing TSP-1 induced a remarkable anti-tumor effect on gastric cancer [18]. However, some kinds of human gastric cell lines, such as MKN1, were resistant to oHSVs. In the current study, we suggested that SOCS3 was downregulated by infection of T-01 in MKN1 cells. As discussed, we focused on SOCS3, and generated a new type of oHSVs expressing SOCS3 (T-SOCS3) and compared the third-generation type of oHSV (T-01) with T-SOCS3 in antitumor effect for human gastric cancer cells. In MKN1 cells, which have low expression of SOCS3 after infection of T-01, T-SOCS3 infection promoted SOCS3 expression, while T-SOCS3 could replicate and induce a more potent oncolysis in comparison with T-01.

Gastric cancer tissue generally has heterogeneity. According to *ex vivo* experimental analysis [29], artificially mechanical and enzymatic tissue processing lead to loss of cellular polarity, degenerated protein profile, and loss of tissue structure. Consequently, this procedure might reflect the cellular condition *ex vivo* regarding viral infection and replication. One of highlights of the present study was therefore the analysis of clinically dissected fresh cancerous samples from gastric cancer patients. Established cancer cell lines are acceptable to differ from initial clinical gastric cancer tissues, so we can obtain a different profile of viral infection and replication in comparison with the gastric cancer cell lines. Some cells may express SOCS3 by infection of oHSVs, others may have downregulated SOCS3. To clarify whether T-SOCS3 defeats heterogeneity of gastric cancer tissue, we recruited collagen gel culture of gastric cancer clinical tissue sample. T-SOCS3 promoted cytotoxicity for gastric cancer tissue more than T-01. T-SOCS3 may therefore be a useful tool for mixed and varied types of gastric cancer cells. Furthermore, given the variation between gastric cancer samples, the results imply that it might be helpful to analyze the tumor before making a choice from OVs for clinical application for oncolytic virotherapy.

Several limitations of this study should be acknowledged. In this study, we used eight different gastric cancer patients’ samples. Among these samples, there were no difference in SOCS3 expression by immunohistochemical staining. However, our experimental method *ex vivo* using freshly dissected gastric cancer tissues was technically difficult to measure pSTAT signal transduction. To clarify the mechanism of a better antitumor effect of T-SOCS3, we need to use tumor-bearing animal model. We have also not clarified the function of SOCS3 in the tumor microenvironmental and systemic immunity. Recent studies indicated that SOCS3 could regulate immune checkpoint molecules to control response to immune cells such as macrophages and T cells [30], for example, the deletion of SOCS3 in T lymphocytes induced antitumor immunity in murine tumor models [31]. On the other hand, inhibition from JAK-STAT3 signaling with SOCS family including SOCS3 induced activation of antitumor immunity in the tumor microenvironment [32]. Antitumor immunity regarding oncolytic virotherapy using T-SOCS3 therefore requires evaluation.

In conclusion, we clarified that an oHSV armed with SOCS3 enhanced the viral replication and oncolysis in human gastric cancer cells, and that the combined effect of SOCS3 with oHSVs inhibited human gastric cancer specimen proliferation *ex vivo*. Further investigation is needed to confirm whether the antitumoral immune reaction mediated by T-SOCS3 treatment can facilitate the therapeutic effect of T-SOCS3 against human gastric cancer.

## MATERIALS AND METHODS

### Cell lines

Africa green monkey kidney cells (Vero) and human gastric cancer cell lines (MKN1, MKN28, and MKN74) were made a purchase from the RIKEN BioResource Center (Tsukuba, Japan). Vero cells were cultivated in Dulbecco’s modified Eagle’s medium (DMEM) with 10% fetal bovine serum (FBS) (Thermo Scientific, USA), and all human gastric cancer cell lines were also cultivated in RPMI-1640 with 10% FBS.

### Oncolytic viruses

T-01 is an HSV-based OV, generated from deletion of the ICP6 gene, α47 gene, and both copies of the γ34.5 gene as previously reported [18]. The viral property of T-01 is the same as G47Δ [25]. To generate an arming oHSV expressing human SOCS3, the oligonucleotide primer sequence for human SOCS3 cDNA was used as follows: 5′-ATGGTCACCCACAGCAAGTT-3′, sense, and 5′-CTTAAAGCGGGGCATCGTACTG-3′, antisense (Takara Bio, Japan). To construct oHSVs, a BAC system and Cre-loxP and FLPe-FRT recombinase method was adopted as previously reported [18, 23, 33]. Particularly, mutagenesis of the T-BAC plasmid was used according to a two-step replacement method. At the first step, human SOCS3 cDNA fragment was ligated into the *StuI-SacII* multicloning site of SV-01 to construct SV-SOCS3. At the second step, after compound of T-BAC plasmid (1.5 μg) and SV-SOCS3 (150 ng) were co-incubated with Cre recombinase (New England BioLabs, MA, USA), the mixture was transformed into competent *Escherichia coli* cell DH10B (Invitrogen, CA, USA) through electroporation. To confirm DNA structures of the recombinant T-BAC/SV-SOCS3, gel analyses following digestion with *Hind III* also performed. At the third step, the ligation mixture of 2 μg of T-BAC/SV-SOCS3 DNA (2 μg) and pOG44 (0.5 μg, Invitrogen) was performed direct transfection into Vero cells with Lipofectamine 2000 and Plus Reagent (Invitrogen). Finally, the progeny viruses were selected and purified by three rounds of limiting dilution. A single clone was sorted out and harvested on Vero cells, and were designated as T-SOCS3. T-SOCS3 viruses were stocked by releasing the virus from infected Vero cells in the presence of heparin, followed by fast-speed centrifugation, as previously reported [18, 33].

### *Ex vivo* immunocytochemical staining

Human gastric cancer cell lines (MKN1, MKN28 and MKN74 cells) were cultured in 6 well plates at 1 × 10^6^ per well. As described previously [18], after 24 hr of incubation, 4% paraformaldehyde/PBS fixed cells. After cells were washed in PBS (–), to block endogenous peroxidase, cells were incubated with 3% hydrogen peroxide in methanol and then cultured in protein blocking solution (Dako Cytomation, Denmark) after rinse in PBS (–). And then, cells were cultured with an anti-human SOCS3 antibody [1:20] (R&D Systems, Minneapolis, MN, USA). After cells were rinsed in PBS (–), they incubated with Histofine Simple Stain MAX (MULTI) (Nichirei, Japan). To detect the immunostaining as a brown product, Diaminobenzidine was used as a chromogen, Finally, strip sections were stained with hematoxylin. Sections and their images were captured by use of a Nikon ECLIPSE 80i microscope (Nikon, Tokyo, Japan).

### Western blotting assay

Vero cells and human gastric cancer cells (MKN1, MKN28, and MKN74) were incubated in 10-cm dishes at 2 × 10^6^ cells per dish at 37°C. After 24 hr of incubation, cells were infected with PBS (–) and T-01 (Vero cells: at MOI of 0.01, human gastric cancer cells: at MOI of 0.1) and T-SOCS3 (Vero cells: at MOI of 0.01, human gastric cancer cells: at MOI of 0.1). After 20 hr of incubation at 39.5°C, proteins (30 μg) were applied to sodium dodecyl sulfate polyacrylamide gel electrophoresis (SDS-PAGE), and then transferred to nitrocellulose membrane (Bio-Rad) and blotted for 2 hr with additive monoclonal mouse anti-SOCS3 antibody (diluted 1:1000, R&D systems), anti-pSTAT3 antibody (1:1000, Cell Signaling Technology, Danvers, MA, USA) or with mouse anti-β-actin antibody (diluted 1:2000, Sigma) for an hour. After the membrane rinsed out, a horseradish peroxidase (HRP)-conjugated anti-mouse secondary antibody (diluted 1:4000, GE healthcare, Piscataway, NJ, USA) was blotted and rinsed. And the, the membrane was exposed to enhanced luminol-based chemiluminescent (ECL) Plus (GE healthcare, Japan).

### *Ex vivo* cell-killing effect of T-01 for gastric cancer cell lines

T-01 was used to confirm cell-killing effect for human gastric cancer cell lines *ex vivo*. MKN1, MKN28, and MKN74 cells were seeded on 24 well plates at 1 × 10^4^ per well. After 24 hr of incubation, the cancer cells were infected through T-01 at an MOI of 0.01 and further incubated at 37°C. By use of a the CytoTox 96 assay (Promega, WI, USA), amount of LDH from gastric cancer cells was measured in triplicate wells. Viable cancer cells were calculated as a percentage of the PBS (–) treated control cells.

### Comparison of viral replication and cell-killing effect of T-01 and T-SOCS3 *ex vivo*

For viral replicative studies, Vero cells and MKN1 gastric cancer cells which are minimally sensitive to T-01, were incubated on 12 well plates at 1 × 10^5^ per well. After 24 hr of incubation, each well was transmitted with either T-01 or T-SOCS3 at MOI of 0.1 (for MKN1 cells) or at MOI of 0.01 (for Vero cells) for 1 hr. After 48 hr of incubation, the cells were scraped off and lysed by three cycles of freezing and thawing. And then, by plaque assay on Vero cells, the viral titer was determined as described [18]. For cell-killing effect studies of T-01 and T-SOCS3, Vero cells and MKN1 cells were incubated on 24 well plates at 1 × 10^4^ per well. After 24 hr of incubation, either T-01 or T-SOCS3 at MOI of 0.1 or 0.01 infected each well. After infection and incubation, the number of viable cancer cells was counted as a percentage of the PBS (–) treated control. Each experiment was measured in triplicate.

### Killing effect of T-01 and T-SOCS3 for gastric cancer tissue specimen in collagen gel culture

To evaluate the antitumor effect of oncolytic HSVs, surgical sections of gastric cancer tissue were cultured in collagen medium for a short time (within 72 h), as previously described [29]. This *ex vivo* experimental study received approval by the Wakayama Medical University Human Ethics Review Committee. Human gastric cancer specimens derived from radical gastrectomy were incubated *ex vivo* on collagen gel immediately after resection. In particular, the mixture of cell matrix type I-A collagen (3 mg/ml; Nitta Gelatin, Japan), reconstitution buffer comprising 2.2% (w/v) NaHCO_3_, 0.2 M HEPES, and 50 mM NaOH, and 10× RPMI-1640 medium was poured into 24-well dishes (0.5 ml/well). After preparation of gastric cancer specimens, 2 mm^3^ cancer pieces placed on collagen gel. Each well was treated with PBS (–), T-01, or T-SCOCS3 at dose of 1 × 10^9^/ml for 1 hr. After 72 hr of incubation at 37°C, the cells viability of gastric cancer tissues was assessed by use of a Cell Titer 96 AQueous One Solution Cell Proliferation Assay (Promega, USA). For H&E staining, virus-treated cancer tissues were embedded in paraffin, and then histological examination was performed according to the instruction manual.

### Statistical analysis

Statistical analysis was calculated by use of Student’s *t* test or one-way ANOVA. All analyses were conducted by use of GraphPad Prism 8 (GraphPad Software, Inc., CA, USA). *P* < 0.05 was considered significant.

## Abbreviations

HSV: herpes simplex virus
SOCS: suppressor of cytokine signaling
TSP-1: thrombospondin-1
JAK: Janus kinase
STAT: signal transducer and activator of transcription
BAC: bacterial artificial chromosome
